# Plasmonic Optical Fiber Sensor Based on Double Step Growth of Gold Nano-Islands

**DOI:** 10.3390/s18041267

**Published:** 2018-04-20

**Authors:** José M. M. M. de Almeida, Helena Vasconcelos, Pedro A. S. Jorge, Luis Coelho

**Affiliations:** 1 CAP/INESC TEC—Technology and Science and FCUP—Faculty of Sciences, University of Porto, 4169-007 Porto, Portugal; hcgv@inesctec.pt (H.V.); pedro.jorge@inesctec.pt (P.A.S.J.); lcoelho@inesctec.pt (L.C.); 2 Department of Physics, School of Sciences and Technology, University of Trás-os-Montes e Alto Douro, 5001-801 Vila Real, Portugal

**Keywords:** optical fiber sensor, localized surface plasmon resonance, gold nanoparticles, gold thin film dewetting

## Abstract

It is presented the fabrication and characterization of optical fiber sensors for refractive index measurement based on localized surface plasmon resonance (LSPR) with gold nano-islands obtained by single and by repeated thermal dewetting of gold thin films. Thin films of gold deposited on silica (SiO_2_) substrates and produced by different experimental conditions were analyzed by Scanning Electron Microscope/Dispersive X-ray Spectroscopy (SEM/EDS) and optical means, allowing identifying and characterizing the formation of nano-islands. The wavelength shift sensitivity to the surrounding refractive index of sensors produced by single and by repeated dewetting is compared. While for the single step dewetting, a wavelength shift sensitivity of ~60 nm/RIU was calculated, for the repeated dewetting, a value of ~186 nm/RIU was obtained, an increase of more than three times. It is expected that through changing the fabrication parameters and using other fiber sensor geometries, higher sensitivities may be achieved, allowing, in addition, for the possibility of tuning the plasmonic frequency.

## 1. Introduction

The measurement of the refractive index (RI) of liquid solutions brings information about its chemical and physical properties, as well as the composition and concentration of dissolved biochemical or biological substances [1]. There are several methods to measure the RI using either free space propagation or guide wave optics [2,3].

Optical fiber sensors (OFS) based on surface plasmon resonance (SPR) can be made with metallic thin layers on top of specific optical fiber surfaces, creating features highly sensitive to the surrounding medium [4,5,6]. In certain conditions, the fabrication of noble metal thin films with nanoscale patterns, usually gold (Au) and silver (Ag), leads to a buildup of the electromagnetic field at the nanostructures boundaries and the phenomenon of localized surface plasmon resonance (LSPR) may take place. This effect results in the appearance of absorption bands whose resonant frequency depends upon the size, shape, and composition of the nanoparticles (NP) and the surrounding RI [7,8,9], allowing label-free real-time RI measurements.

Several protocols have been developed to obtain Au NP with the chosen size and shape, dispersion stability and surface functionality. These methods can be classified either as physical or chemical [10,11,12,13,14]. However, while the physical methods require expensive equipment, the chemical methods are laborious and often lead to non-uniform films. The thermal annealing of metal thin films such as Au [15], Ag [16] and palladium [17], results in the formation of nano-islands on the surface (dewetting), offering a low-cost technique, with potential large-scale fabrication capability to produce plasmonic sensors with LSPR ability. The dewetting of Au thin films has been the object of studies on the influence of the thin film deposition conditions, substrate preparation, and thermal annealing parameters on the morphology of the nano-islands and on the characteristics of the plasmonic resonance [18,19,20]. Recently it was reported by Kang, M. [21], that repeating the standard thermal dewetting process of Au thin films, nano gap rich Au islands are obtained. The method provides enlarged Au nano-islands with small gap spacing, which increases the number of electromagnetic hotspots and thus enhances the extinction intensity as well as the tunability for plasmon resonance wavelength.

Optical fiber sensors based on LSPR produced by dewetting of Au thin films were produced in unclad fiber [22] and, recently, a 50/3/50 μm hetero-core structured fiber coated with Au nano-islands around the cladding was demonstrated with wavelength sensitivity of 517 nm/RIU [23]. The optimization of OFS coated with Au thin film and annealed was presented [24]. The multimode fiber was immersed in piranha solution and then subjected to a lengthy silanization process for improving the Au adhesion to the silica core. When an SPR zone of 6 mm in length was built, a sensitivity higher than 700 nm/RIU was measured.

The objective of the study presented here is the comparison of the performance of OFS where the plasmonic region is made either by the single or by the repeated thermal dewetting of Au thin film deposited only at the end of multimode fibers. In this way, an extra degree of freedom is introduced in the design of OFS/LSPR based on Au dewetting.

## 2. Materials and Methods

### 2.1. Sample Fabrication

The fiber sensors were prepared using multimode fiber (FT600UMT Thorlabs, Dortmund, Germany) with a diameter of 600 μm which was split into fragments with a length of ~4 cm. The plastic fiber jacket was mechanically detached from the fiber and the TECS Hard Fluoropolymer Cladding (not compatible with the annealing temperatures) removed by dissolving in acetone to expose the silica core. Both ends were polished using 8 and 3 μm polishing disks (Fibrmet, Buehler, Lake Bluff, IL, USA) until an optical surface was reached. In addition, a set of silica planar substrates was used to fabricate and characterize nano-structured Au thin films using a broad range of conditions.

Prior to deposition of Au, silica planar substrates and optical fibers were placed in a Teflon sample holder and washed in a mixture of detergent (Decon 90) with deionized water in an ultrasonic bath at 30 °C for 10 min. Further, the samples were placed in acetone, methanol, and water following the same procedure. Then the samples were dried under a N_2_ stream and dried in an oven at 100 °C for 10 min.

For the deposition of Au, two metal holders were designed to hold the silica substrates and the fibers (with the end faces facing down). The holders were fixed onto a circular metal plate with a diameter of ~200 mm. The Au thin films were deposited using an electron beam evaporator model Auto 306 (Edwards, Ltd., Bolton, UK). A thickness monitor model FTM5 (Edwards, Ltd., UK) provides measurements with a resolution of 0.1 nm. The thickness of the Au thin films ranged from 2 to 12 nm and the adhesion to the substrate was improved with a 2 nm buffer layer of chromium (Cr).

For the thermal dewetting of the Au thin films, the samples were placed in the central region of a 2500 W tubular furnace (Carbolite, UK) for 1 h at 500, 550, 600 and 650 °C, respectively. The temperature of the oven was raised at a rate of ~10 °C per minute and the cooling process took place at the same rate. The indicated annealing time was counted after the sample reached the chosen temperature until it starts to cool down.

### 2.2. Sample Characterization

Gold nano-islands formation on silica planar substrates was characterized by Scanning Electron Microscope (SEM) and by Energy Dispersive X-ray Spectroscopy (EDS). The SEM illustrates the topologic changes due to thermal annealing, while EDS, being a semi-quantitative technique, may be used to confirm the presence of gaps between the Au nano-islands.

**Figure 1:**
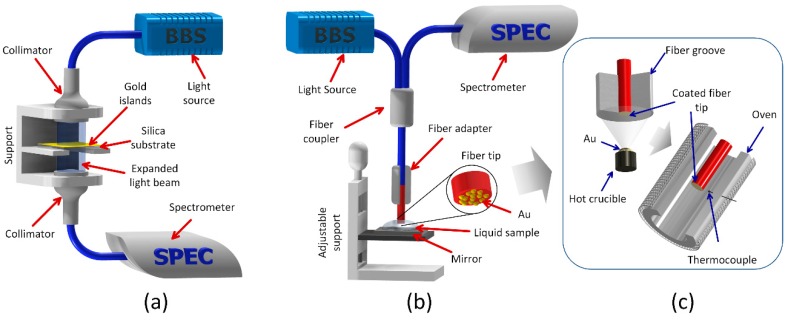
(**a**) Setup for the measurement of the absorption spectra in transmission mode of planar silica samples coated with gold nano-islands, (**b**) Setup for measurement in reflection mode of refractive index sensitivity of optical fibers coated with gold nano-islands, (**c**) Set-up for coating with Au only the end surface of the fibers; after Au coating, annealing took place in an oven.

The absorption spectra of planar samples were measured in transmission mode, using the setup illustrated in Figure 1a, from the ultraviolet (~300 nm) to near infrared (~900 nm), and were acquired with a resolution of 1 nm and an integration time of 100 ms. A tungsten halogen light source equipped with an SMA905 fiber connector was used (Avantes, AvaLight-Hal, Apeldoorn, The Netherlands). The normalized spectra were collected and recorded as an absorbance (in decibels) using a diode-array fiber optic spectrometer (Speed+, SarSpec, Porto, Portugal). The light from the fiber cable was collimated by a lens and after it passes through the planar sample was collected by a second lens, where a second fiber cable connects to the spectrometer.

The optical characterization of the fiber samples was performed in reflection mode using the setup illustrated in Figure 1b which includes a bifurcated fiber cable (SPLIT400-VIS-NIR, Ocean Optics, Largo, FL, USA) to connect the 4 cm long fiber sensors to the light source and spectrometer. The fibers were placed in a holder that enables coating with Au only the end surface, Figure 1c, and after that, the thin films were annealed. The wavelength sensitivity of the fiber samples was determined by varying the surrounding refractive index (SRI) from 1.300 to 1.640 using a set of calibrated oils (Cargille–Sacher Laboratories, Inc., Cedar Grove, NJ, USA). The oil sample to be tested was placed in a plane mirror and the fiber sensor lowered until its tip was immersed in the oil drop by means of a translation stage. After each measurement, the fiber and the mirror were flushed with acetone to avoid contamination to the next oil sample.

A one-tailed *t*-test was implemented to determine if two sets of samples have particle diameter significantly different from each other. To verify whether the mean particle diameter of different groups are equal, the analysis of variance (ANOVA) was applied [25].

## 3. Experimental Results and Discussion

### 3.1. SEM/EDS Characterization of Planar Samples

**Figure 2:**
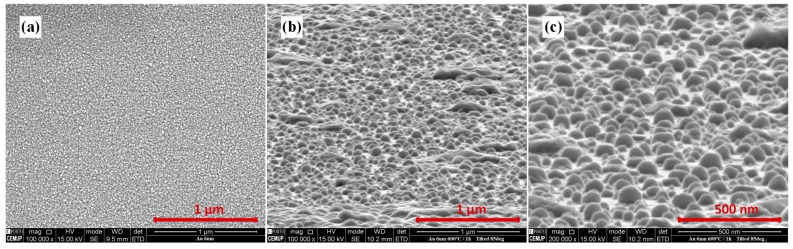
Scanning Electron Microscope (SEM) photograph of a sample coated with 6 nm thick gold thin film: (**a**) before and (**b**,**c**) after annealing at 600 °C for 1 h.

Silica substrates coated with Au were analyzed by SEM/EDS techniques before and after the annealing process. Figure 2 presents a sample coated with 6 nm thin film, (a) before and both (b) and (c) after annealing at 600 °C for 1 h. The photographs confirm that the thin films were converted to a discontinuous nano island structure after annealing.

**Figure 3:**
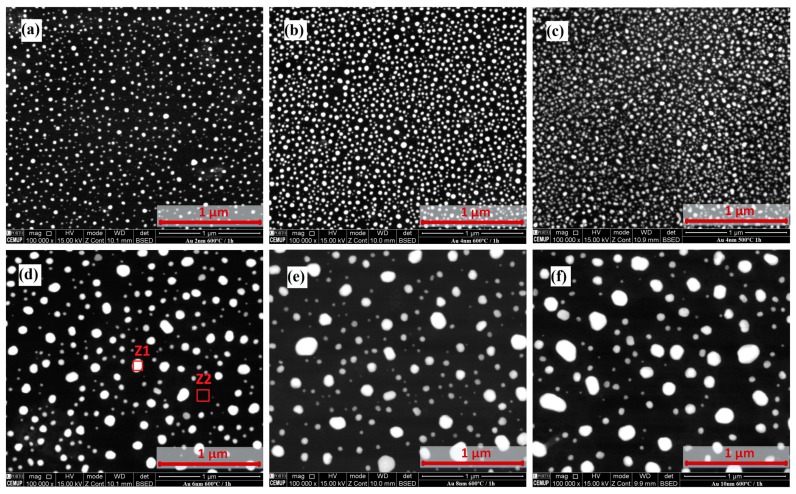
Scanning Electron Microscope (SEM) photograph of samples annealed at 500 and 600 °C for 1 h for 2 to 10 nm tick Au thin films: (**a**) 2 nm/600 °C, (**b**) 4 nm/600 °C, (**c**) 4 nm/500 °C, (**d**) 6 nm/600 °C, (**e**) 8 nm/600 °C and (**f**) 10 nm/600 °C. The marked regions Z1 and Z2 on (**d**) were analyzed by Energy Dispersive X-ray Spectroscopy (EDS).

Figure 3 shows the SEM photographs (taken with the built-in backscattered electron detector) of samples annealed at 500 and 600 °C for 1 h and coated with 2 to 10 nm Au thin films: (a) 2 nm/600 °C, (b) 4 nm/600 °C, (c) 4 nm/500 °C, (d) 6 nm/600 °C, (e) 8 nm/600 °C and (f) 10 nm/600 °C. Distinct regions Z1 and Z2 marked on Figure 3d were analyzed by EDS allowing to estimate the composition of the surface inhomogeneity at different locations. The same is observed in all the samples. The average particle diameter after annealing was calculated using the image analysis software ImageJ (National Institutes of Health, Bethesda, MD, USA). The average diameter of the nano-islands for samples with the starting thickness of 2, 4, 6, 8 and 10 nm is presented in Table 1. From the ANOVA test it may be concluded that the 2 and 4 nm thin film samples are not significantly different, as well as the 8 and 10 nm samples. It can be observed that the island diameter increases with the film thickness. The gap between the different nano-islands seems to be similar to the island size. However, no quantitative validation was performed. There is a steady decrease in the island surface density with the increase of the film thickness, although, analysis of Figure 3b,c led to the conclusion that it is not affected by the annealing temperature.

**Figure 4:**
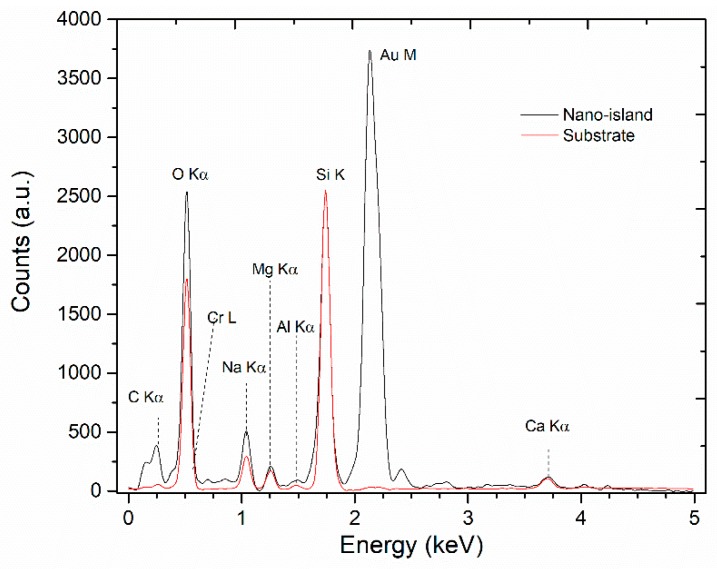
Energy Dispersive X-ray Spectroscopy (EDS) analysis of a 6 nm sample after annealing at 600 °C for 1 h.

The EDS analysis of a 6 nm sample after annealing is shown in Figure 4. The surface of the sample does not possess a significant number of impurities. Both spectra show the well-defined band at around 1740 eV corresponding to the Kα_1_, Kα_2_ and Kβ_1_ lines of the Si K band. The adhesion layer band Cr L is overlapped by the wings of the larger oxygen line (O Kα) and a band related to carbon contamination (band C K) is present. The band related to Au is present in region Z1 (nano-island) but not in region Z2 (substrate). Therefore, the white spots correspond to pure Au nano-islands, which is not present in remaining surface, meaning that the Au thin film undergoes nucleation after thermal treatment.

### 3.2. Optical Characterization of Planar Samples

The optic/plasmonic properties of noble metal nanostructures depends on the nanoparticle size and shape and distance between particles [9,19]. The morphology of the nano-structured films can be modified by controlling the initial film thickness, the temperature and the time of annealing. In addition, the deposition rate of the initial thin film, the annealing atmosphere pressure and its composition play an important role [18]. The present work shows the influence of the initial Au film thickness and the annealing temperature on the LSPR characteristics.

**Figure 5:**
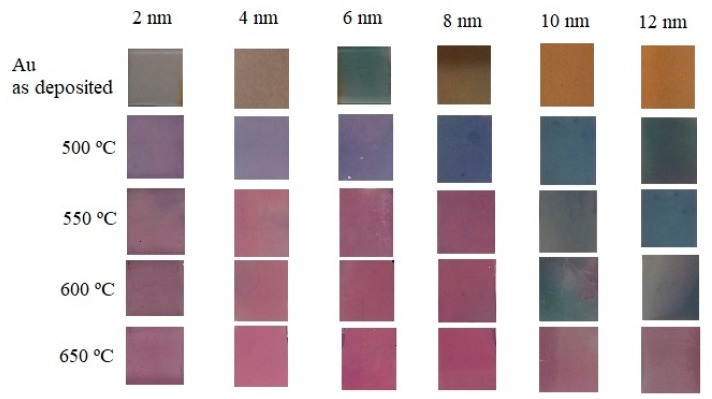
Gold coated planar samples photographed before and after annealing at 500, 550, 600 and 650 °C for 1 h with initial thicknesses of 2, 4, 6, 8, 10 and 12 nm.

The Au coated planar samples were photographed before and after the thermal treatment with a digital camera (7 megapixel, Sony Cyber-shot). Before the thermal treatment, the Au films exhibit different colors changing from light gray for 2 nm to grayish-blue for 6 nm and to yellowish golden color for 12 nm thick films, as illustrated in Figure 5. After annealing for 1 h, the colors of the planar samples have changed. For the 2 nm sample a change can be already observed at 500 °C, while the 4 to 8 nm samples turn violet at 550 °C. Thicker samples change color only at 650 °C. As can be seen in Figure 5, higher annealing temperature leads to a dark pink/violet color.

**Figure 6:**
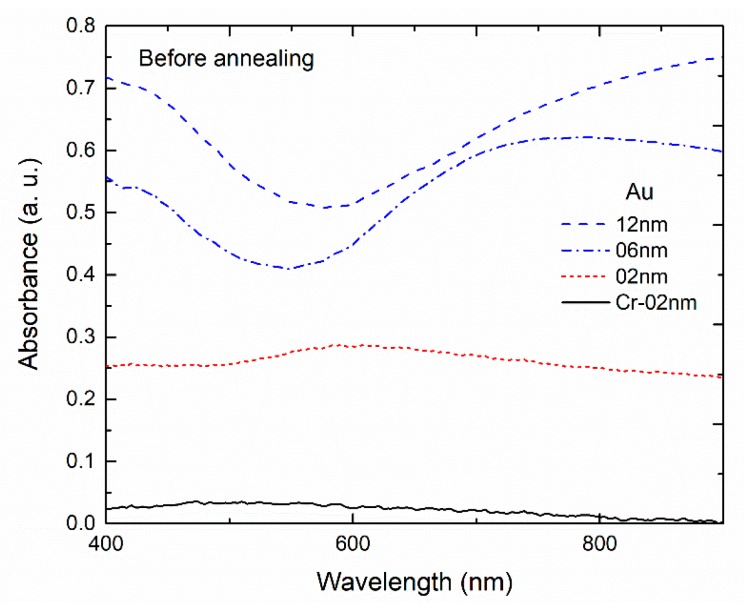
Absorption spectra before annealing of planar samples with 2, 6 and 12 nm gold thin film deposited on substrates with 2 nm of chromium (adhesion layer) and of a sample with 2 nm chromium thin film.

The absorption spectra before annealing of samples with 2 nm of Cr (adhesion layer), and 2, 6 and 12 nm thick Au film deposited are shown in Figure 6. The absorption spectrum of the sample with only the Cr layer is characterized by a flat band and very small absorbance compared to the samples with Cr and Au. It can be concluded that the Cr adhesion layer has low effect on the optical spectroscopy. The sample with 2 nm of Au, shows a plasmonic broad absorption band with a peak at ~600 nm. For samples with higher Au film thickness, the absorption bands exhibit similar characteristics, with a minimum below 600 nm followed by a smooth band for higher wavelengths.

The wide absorption band for 2 nm Au sample may be ascribed to the formation of nano-structures during the well-known process of thin films growth. In gold thin film growth considering the Volmer-Weber mode [26], isolated nano-islands are initially formed that nucleate and coalesce each other [21]. The spectral characteristics of 6 to 12 nm samples are characteristic of semi-continuous plasmonic thin films [18,27].

**Figure 7:**
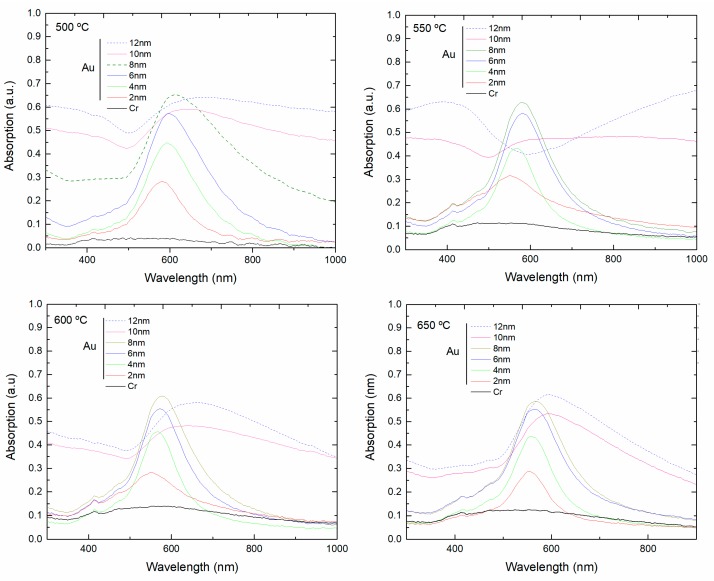
Absorption spectra after annealing at 500, 550, 600 and 650 °C for 1 h of planar samples with 2, 4, 6, 8, 10 and 12 nm gold thin film deposited on substrates with 2 nm of chromium (adhesion layer) and of a sample with 2 nm chromium thin film.

**Figure 8:**
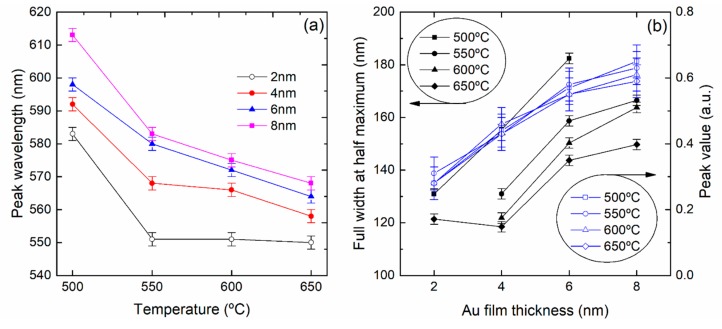
Evolution of the plasmonic band as a function of the annealing temperature for 2, 4, 6 and 8 nm gold thin film: (**a**) peak wavelength and (**b**) full width at half maximum and peak intensity.

As shown in Figure 7, the absorption spectra of samples with different Au thickness changed significantly after annealing at 500, 550, 600 and 650 °C for 1 h. Figure 8a,b summarizes the variation of the peak wavelength, extinction intensity and full width at half maximum (FWHM) with the annealing temperature and thin film thickness. At 500 °C the thinner samples (2 to 6 nm) already show well-defined plasmonic bands, while the extinction value increases for thicker Au thin films. At 550, 600 and 650 °C the plasmonic band is now present in the 8 nm thick samples. The thicker 10 and 12 nm samples retain the initial features of a continuous thin film, not presenting plasmonic features at 550 and 600 °C, but at 650 °C a broad band is now observed, indicating the formation of nano-islands. For 2 nm of Au the resonant band has a peak at 582 nm, and increasing the Au thickness to 4 and 6 nm led to a red shift, to 593 and 598 nm, respectively. The redshift and the increasing of the extinction value for thicker Au films is patent. As shown in Figure 8a,b, as the temperature increases, for a given film thickness, the absorption band moves to shorter wavelengths and becomes narrower. Within the experimental error, the extinction intensity is not affected by the annealing temperature, but only by the film thickness.

### 3.3. Repeated Thermal Dewetting of Au Thin Film in Planar Samples

The optical properties of metallic thin films depend on the dielectric constant of the surrounding medium (ε_m_). For nano-islands structured thin films, the effective dielectric constant is a function of the volume fraction, *F*, (number of nano-islands times the volume of each nano-island) and the polarization of the nano-islands which is proportional to the *F* value [28]. Based on the free electron model for metallic structures, the maximum absorption of the LSPR (*λ_m_*) and the wavelength of the plasmon peak of bulk metal, *λp* (for Au, *λp* = 131 nm) is related with *F* and with the refractive index of the surrounding medium(*n_s_*) by Equation (1) [29]:

(1) $$\lambda_{m}=\lambda_{p}{\left[1+\left(\frac{2+F}{1-F}\right)n_{s}^{2}\right]}^{1/2}$$

For a given *n_s_* value, the maximum absorption increases with *F* and the LSPR peak shifts to higher wavelengths when *n_s_* increases.

A method for obtaining Au nano-islands with high density of nano-gaps for highly sensitive surface-enhanced Raman scattering (SERS) was reported [21]. The method consists of repeating the thermal dewetting process of Au thin films on the same substrate, providing enlarged gold nano-islands with small gap spacing. The result is an increase of the electromagnetic hotspots leading to an increase of the extinction intensity as well as the tunability for plasmon resonance wavelength.

Repeated dewetting was accomplished in first place on a set of planar silica substrates by alternating Au thin film evaporation and thermal annealing, as reported in [21]. The first step consists in the thermal evaporation of Au thin film on silica substrates coated with 2 nm Cr followed by thermal annealing to obtain Au nano-islands. In the second step, a second Au thin film was deposited on the substrate with Au nano-islands obtained in the previous step and then annealed, following the same conditions.

Gold thin films with 6 nm thickness were deposited and annealed at 600 °C and the process (deposition and annealing) repeated. Simultaneously, for comparison, a set of samples was prepared by single dewetting, using 6 and 12 nm thick Au films.

**Figure 9:**
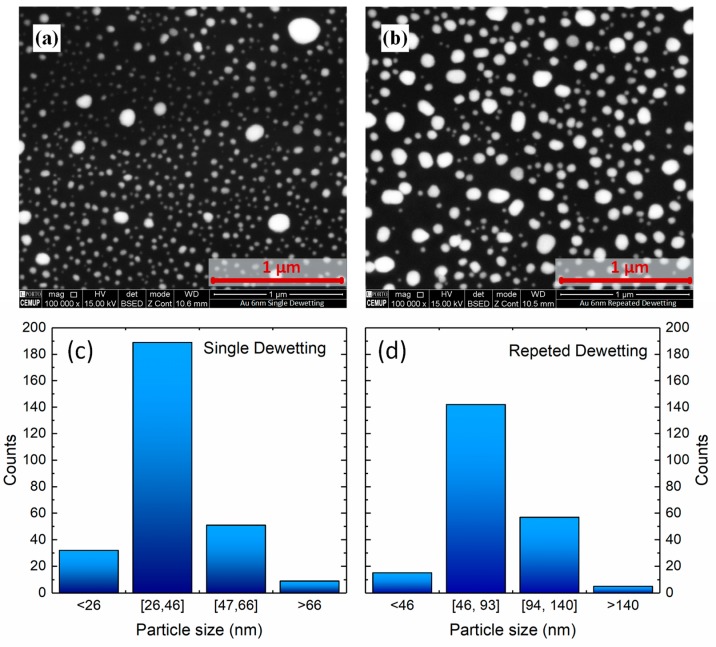
Scanning Electron Microscope photographs of gold nano-islands under different dewetting conditions of 6 nm thick gold film: (**a**) single step and (**b**) repeated dewetting. Plots (**c**,**d**), are calculated showing distribution of particle size, for samples shown in (**a**,**b**), respectively.

Figure 9 shows SEM images of Au nano-islands under different dewetting conditions: (a) single step and (b) repeated dewetting. The images show apparently engorged Au nano-islands and smaller spacing after the repeated dewetting, compared to the single-step sample. The distribution of particle size for single or repeated dewetting, calculated with the software ImageJ software, shows that using the single dewetting process ~66% of the particles have sizes between 26 and 46 nm, while for the repeated dewetting the same percentage is fitted between 46 and 93 nm as shown in Figure 9.

The repeated dewetting process exhibits higher packing density of nano-islands with an Area Fraction of 18.5%, due to the enlarged effective diameter, compared to the single-step dewetting which possesses an Area Fraction of 11.3%. A one-tailed *t*-test was performed and a *p*-value of 0.036 was achieved, meaning that the difference is statistically significant.

The nano-structured thin film developed initially, by forming isolate nano-islands, nucleate and merge during the first step, and according to [21], during the second dewetting, the nano-islands suffer rapid coalescence, rather than nucleation. Consequently, the second dewetting gives rise to an increase of the size of the nano-islands formed in the first step and a reduction of the gap between nano-islands takes place.

**Figure 10:**
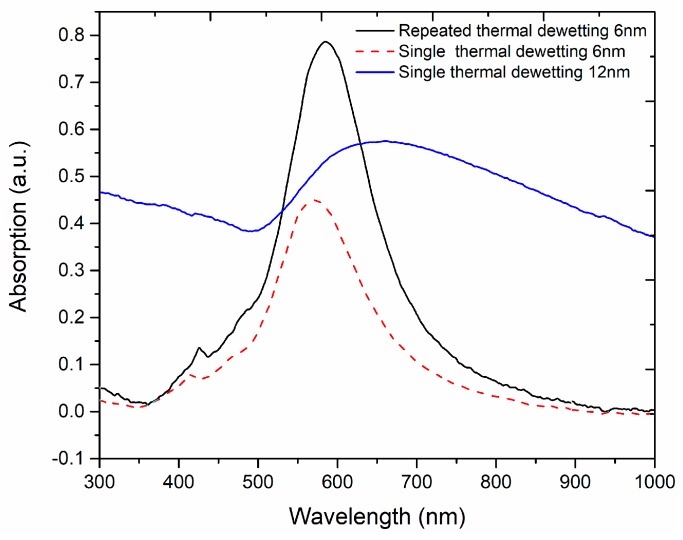
Absorption spectra of nanostructures in planar samples prepared by single step and repeated dewetting of 6 nm thick gold film and of a sample prepared by single step dewetting of an Au film 12 nm thick film.

For an Au film 6 nm thick, the extinction spectra shown in Figure 10, for single step and repeated dewetting, reveal that larger nano-islands with small gap augments the absorption and led to a shift of the band to higher wavelength (~12 nm). These results are compatible with theoretical and experimental published studies on the optimization of refractive index sensitivity of plasmonic coupled Au nanoparticles [30]. An increase of the size of the nano-islands cause a red shift of the extinction band. In addition, the peak of the plasmon resonance depends not only on the size but also on the distance between the nano-particles [27]. When nano-islands are brought closer to each other, their oscillations interact resulting in optical coupling, which results in red-shifted resonance modes.

The absorption spectrum of a sample prepared by single step deposition and annealing of an Au film 12 nm thick is also shown in Figure 10, where it can be observed that the spectral features are characteristic of semi-continuous plasmonic thin films. Therefore, the repeated dewetting method plays an effective role in enhancing the extinction band.

### 3.4. Optical Characterization of Fiber Sensors

The edge surface of the core of a set of multimode silica optical fibers was coated with 6 and 8 nm thick of Au film and annealed at 600 °C for 1 h. A sub set of the 6 nm coated fibers was re-coated after annealing with another 6 nm thick Au film and annealed again at 600 °C for 1 h.

**Figure 11:**
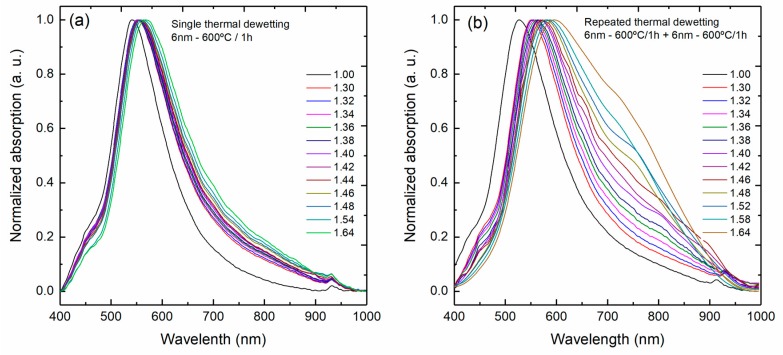
Evolution of the absorption spectra from 400 to 1000 nm as a function of the refractive index of the surrounding medium from 1.30 to 1.64 of multimode silica optical fibers coated with a 6 nm of gold and fabricated at 600 °C for 1 h by (**a**) single step and (**b**) repeated dewetting.

Using the set up illustrated in Figure 1b, the absorption spectra of these fibers were measured for refractive index values of the surrounding medium from 1.30 up to 1.64, as shown in Figure 11a,b for the single step and for the repeated dewetting fibers, respectively.

**Figure 12:**
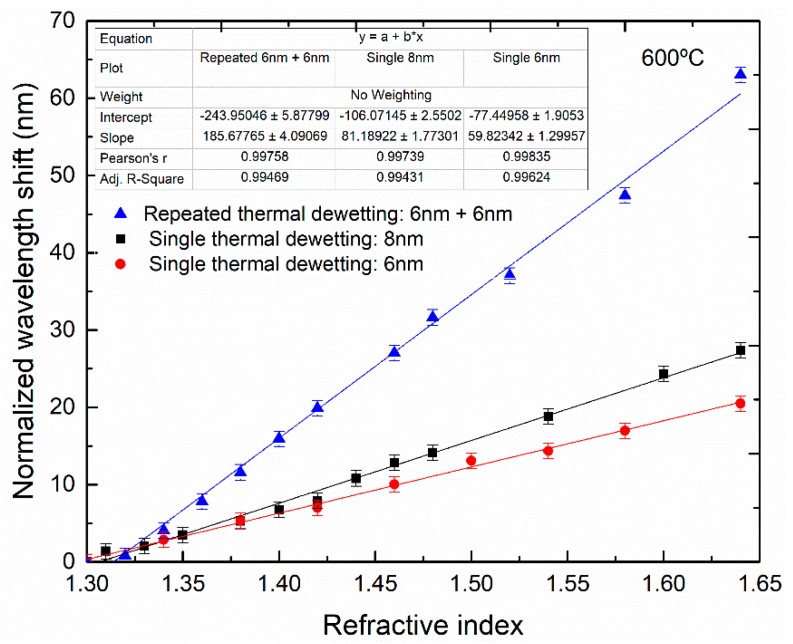
Calculated normalized wavelength shift as a function of the surrounding refractive index from 1.30 to 1.64 of multimode silica optical fibers coated with a 6 and 8 nm of gold and fabricated at 600 °C for 1 h by single step (6 and 8 nm) and repeated dewetting (6 nm).

The calculated normalized wavelength shift as a function of the surrounding refractive index, from 1.30 to 1.64 is presented in Figure 12. The sensors presented here are meant to be used to monitor liquid mixtures in the range 1.33 to 1.40; therefore, normalization of the wavelength shift was performed using, for each fiber, the peak wavelength at 1.30. The sensitivity to the surrounding refractive index was calculated from Figure 12 and for the 6 and 8 nm single step dewetting the values of 59.8 and 81.2 nm/RIU was achieved, respectively. Using the repeated dewetting process with 6 nm thick Au film a higher sensitivity of 185.7 nm/RIU was reached for the same refractive index range, a 3-fold improvement when compared to the single process.

The refractive index sensitivity changes with the size and shape of the nano-structures, but also with their interaction. The higher sensitivity for the 8 nm thick Au film compared with 6 nm thick may be explained by the larger particle size of the former. Regarding the repeated dewetting samples, the increase in sensitivity may be attributed to the higher particle size but also to the decrease in gap spacing.

## 4. Conclusions

This work reports the application of a simple fabrication method for obtaining nano-gap rich gold nano-islands with enlarged sizes based on repeated deposition and thermal annealing of gold thin film. These nano structures are meant to be used in the development of highly sensitive refractive index optical fiber sensors. A large increase in sensitivity was obtained over the single step dewetting process, even though only the core end surface of the optical fiber was coated with gold (600 μm in diameter).

It is expected that the initial and additional gold thin film thickness, the size, gap spacing, packing density, and optical properties may be manipulated in order to design and fabricate specific optical fiber sensors.

## Figures and Tables

**Table 1:** Average diameter of the nano-islands for samples with 2, 4, 6, 8 and 10 nm.

Nano-islands	Thickness of Gold Thin Film (nm)				
	2	4	6	8	10
Diameter ± standard deviation	24.1 ± 5.1 ^a^	30.5 ± 5.3 ^a^	41.9 ± 6.1 ^b^	61.8 ± 12.3 ^c^	73.4 ± 15.2 ^c^

Averages that have different lowercase letters are significantly different (*p* < 0.05).

## References

[1] Pospíšilová M., Kuncová G., Trögl J. (2015). Fiber-optic chemical sensors and fiber-optic bio-sensors. Sensors.

[2] Nath J.P., Singh H.K., Bezboruah T. (2016). Fiber optic refractometers: A brief qualitative review. Adv. Res. Electr. Electron. Eng..

[3] Bhatia P., Gupta B.D. (2011). Surface-plasmon-resonance-based fiber-optic refractive index sensor: Sensitivity enhancement. Appl. Opt..

[4] Jatschka J., Dathe A., Csáki A., Fritzsche W., Stranik O. (2016). Propagating and localized surface plasmon resonance sensing—A critical comparison based on measurements and theory. Sens. Bio-Sens. Res..

[5] Klantsataya E., Jia P., Ebendorff-Heidepriem H., Monro T.M., François A. (2016). Plasmonic fiber optic refractometric sensors: From conventional architectures to recent design trends. Sensors.

[6] Mishra S.K., Varshney C., Gupta B.D. (2013). Surface plasmon resonance based fiber optic refractive index sensor utilizing cu/zno layer. AIP Conf. Proc..

[7] Chung T., Lee S.-Y., Song E.Y., Chun H., Lee B. (2011). Plasmonic nanostructures for nano-scale bio-sensing. Sensors.

[8] Mayer K.M., Hafner J.H. (2011). Localized surface plasmon resonance sensors. Chem. Rev..

[9] Petryayeva E., Krull U.J. (2011). Localized surface plasmon resonance: Nanostructures, bioassays and biosensing—A review. Anal. Chim. Acta.

[10] Saha K., Agasti S.S., Kim C., Li X., Rotello V.M. (2012). Gold nanoparticles in chemical and biological sensing. Chem. Rev..

[11] Grzelczak M., Pérez-Juste J., Mulvaney P., Liz-Marzán L.M. (2008). Shape control in gold nanoparticle synthesis. Chem. Soc. Rev..

[12] Wei J., Zeng Z., Lin Y., Rasooly A., Prickril B. (2017). Localized surface plasmon resonance (lspr)-coupled fiber-optic nanoprobe for the detection of protein biomarkers. Biosensors and Biodetection: Methods and Protocols Volume 1: Optical-Based Detectors.

[13] Sobhan M.A., Withford M.J., Goldys E.M. (2009). Enhanced stability of gold colloids produced by femtosecond laser synthesis in aqueous solution of ctab. Langmuir.

[14] Reed J.A., Cook A., Halaas D.J., Parazzoli P., Robinson A., Matula T.J., Grieser F. (2003). The effects of microgravity on nanoparticle size distributions generated by the ultrasonic reduction of an aqueous gold-chloride solution. Ultrason. Sonochem..

[15] Tesler A.B., Maoz B.M., Feldman Y., Vaskevich A., Rubinstein I. (2013). Solid-state thermal dewetting of just-percolated gold films evaporated on glass: Development of the morphology and optical properties. J. Phys. Chem. C.

[16] Samavat F., Rahman J.J. (2015). Preparation of silver thin films, and the study of the annealing effects on their structures and optical properties. Surf. Topogr. Metrol. Prop..

[17] Kracker M., Worsch C., Rüssel C. (2013). Optical properties of palladium nanoparticles under exposure of hydrogen and inert gas prepared by dewetting synthesis of thin-sputtered layers. J. Nanopart. Res..

[18] Jia K., Bijeon J.-L., Adam P.-M., Ionescu R.E. (2013). Large scale fabrication of gold nano-structured substrates via high temperature annealing and their direct use for the lspr detection of atrazine. Plasmonics.

[19] Gupta G., Tanaka D., Ito Y., Shibata D., Shimojo M., Furuya K., Mitsui K., Kajikawa K. (2008). Absorption spectroscopy of gold nanoisland films: Optical and structural characterization. Nanotechnology.

[20] Al-Rubaye A.G., Nabok A., Tsargorodska A. (2017). Spectroscopic ellipsometry study of gold nanostructures for lspr bio-sensing applications. Sens. Bio-Sens. Res..

[21] Kang M., Park S.-G., Jeong K.-H. (2015). Repeated solid-state dewetting of thin gold films for nanogap-rich plasmonic nanoislands. Sci. Rep..

[22] Meriaudeau F., Downey T., Wig A., Passian A., Buncick M., Ferrell T.L. (1999). Fiber optic sensor based on gold island plasmon resonance. Sens. Actuators B Chem..

[23] Hosoki A., Nishiyama M., Watanabe K. (2017). Localized surface plasmon sensor based on gold island films using a hetero-core structured optical fiber. Appl. Opt..

[24] Antohe I., Schouteden K., Goos P., Delport F., Spasic D., Lammertyn J. (2016). Thermal annealing of gold coated fiber optic surfaces for improved plasmonic biosensing. Sens. Actuators B Chem..

[25] Dalgaard P. (2008). Introductory Statistics with R.

[26] Levine J.R., Cohen J., Chung Y. (1991). Thin film island growth kinetics: A grazing incidence small angle x-ray scattering study of gold on glass. Surf. Sci..

[27] Karakouz T., Maoz B.M., Lando G., Vaskevich A., Rubinstein I. (2011). Stabilization of gold nanoparticle films on glass by thermal embedding. ACS Appl. Mater. Interfaces.

[28] Treu J.I. (1976). Mie scattering, maxwell garnett theory, and the giaever immunology slide. Appl. Opt..

[29] Rai V.N., Srivastava A.K., Mukherjee C., Deb S.K. (2014). Localized surface plasmon resonance (lspr) and refractive index sensitivity of vacuum evaporated nanostructured gold thin films. arXiv.

[30] Martinsson E., Sepulveda B., Chen P., Elfwing A., Liedberg B., Aili D. (2014). Optimizing the refractive index sensitivity of plasmonically coupled gold nanoparticles. Plasmonics.

